# Detection of dengue viruses using reverse transcription-loop-mediated isothermal amplification

**DOI:** 10.1186/1471-2334-13-387

**Published:** 2013-08-21

**Authors:** Boon-Teong Teoh, Sing-Sin Sam, Kim-Kee Tan, Jefree Johari, Mohammed Bashar Danlami, Poh-Sim Hooi, Rafi Md-Esa, Sazaly AbuBakar

**Affiliations:** 1 Tropical Infectious Diseases Research and Education Centre (TIDREC), Department of Medical Microbiology, Faculty of Medicine, University of Malaya, Kuala Lumpur 50603, Malaysia

## Abstract

**Background:**

Early and rapid detection of dengue virus (DENV) infection during the febrile period is crucial for proper patient management and prevention of disease spread. An easy to perform and highly sensitive method is needed for routine implementation especially in the resource-limited rural healthcare settings where dengue is endemic.

**Methods:**

A single-tube reverse transcription-loop-mediated isothermal amplification (RT-LAMP) assay with a set of nine primers was developed for the detection of all four DENV serotypes and their different genotypes. The sensitivity and specificity of the RT-LAMP were evaluated. The clinical applicability of RT-LAMP assay for detection of DENV RNA was assessed in a total of 305 sera of clinically-suspected dengue patients. The test results of RT-LAMP were statistically compared to those of quantitative reverse transcription-polymerase chain reaction (qRT-PCR), IgM- and IgG-capture enzyme-linked immunosorbent assays (ELISA).

**Results:**

Acute DENV infection was confirmed in 171 samples (n = 305); 43.3% (74/171) and 46.8% (80/171) of the samples were positive for DENV using RT-LAMP and qRT-PCR, respectively. The combination of RT-LAMP with the dengue IgM and IgG ELISA increased detection of acute DENV infection to 97.7% (167/171), in comparison to only 70.8% (121/171) when dengue IgM and IgG ELISA alone were used. The RT-LAMP assays showed high concordance (κ = 0.939) with the qRT-PCR. The RT-LAMP assay detected up to 10 copies of virus RNA within an hour but 100% reproducibility (12/12) was achieved with 100 copies. There was no cross reactivity of RT-LAMP with other closely related arboviruses.

**Conclusion:**

The RT-LAMP assay developed in this study is sensitive, specific and simple to perform. The assay improved the detection of dengue when used in combination with serological methods.

## Background

Dengue virus (DENV) is a positive-sense single-stranded RNA virus with a genome of ~10.7 kb in length [1]. There are four antigenically distinct DENV serotypes; DENV-1, DENV-2, DENV-3 and DENV-4 [2] and each serotype contains phylogenetically distinct genotypes [3]. Infection with any of the four serotypes produces a spectrum of clinical illness ranging from mild dengue fever (DF) to severe and fatal dengue hemorrhagic fever (DHF) and hemorrhagic shock syndrome (DSS) [4]. Infection with one serotype leads to lifelong protection against homotypic reinfection but only temporary cross-protection against heterotypic infection [5]. Studies have shown that heterotypic secondary infection is associated with higher risk of contracting DHF and DSS [6] possibly through antibody-dependent enhancement [7,8], original antigenic sin [9,10], cytokine storm [11], or autoimmune response [12,13]. Currently, an estimated 3.6 billion persons living in dengue-endemic countries are at risk of contracting dengue [14]. Viremic individuals are the main source of infectious virus. Virus is transmitted following mosquito bites of these viremic individuals. A sensitive, easy to perform and rapid method for detection of DENV in viremic febrile patients is therefore of paramount importance especially for patient management and immediate vector control measures to prevent large-scale epidemic [15].

Routine laboratory diagnosis of DENV infection in most resource-limited countries is based on the detection of virus-specific antibodies as well as virus isolation. Detection of viral components such as non-structural protein 1 (NS1) and virus genomic RNA are done but mostly in referral or in more well-funded diagnostic laboratories [16-18]. Although virus isolation in C6/36 mosquito cell line has been considered as the “gold standard” for DENV detection, it is costly and time-consuming as more than 7 days is required to complete the assay [19,20]. IgM- and IgG-capture enzyme-linked immunosorbent assays (ELISA) are the most widely used methods to infer DENV infection serologically [21]. However, it requires second convalescent sera for confirmation of test results. It is also not useful during the febrile viremic phase of the infection when there is yet no significant rise in the antibody titers. In addition, antibody cross-reactivity towards other closely related flaviviruses is a common problem [16]. DENV NS1 ELISA, the other known dengue test, is highly sensitive and specific [22,23] but it can be compromised by pre-existing NS1-IgG immunocomplexes in the acute stage of secondary DENV infection, which is common in dengue endemic region [24-27]. More recently, molecular techniques to detect virus genomic RNA sequence by reverse transcription-polymerase chain reaction (RT-PCR) and real-time quantitative RT-PCR (qRT-PCR) are gradually being accepted as new standards over virus isolation for the detection of DENV in acute sera [28-32]. The method is highly useful for detection of virus in viremic individuals. The requirement of costly nucleic acid amplification instrument and high level of skill, however, has restricted the application of these methods especially in resource-limited rural healthcare settings.

The loop-mediated isothermal amplification (LAMP) of genomic sequence is a novel method for the detection of nucleic acid with high specificity and sensitivity without the need of specialized equipment [33-35]. The method requires only a heating block or water bath that can maintain constant temperature between 60 to 65°C as the nucleic acid amplification reaction can be performed at a single constant temperature. It has been described for the detection of various infectious agents including DENV [36-38]. These earlier reports, however, evaluated their RT-LAMP assays for the detection of DENV infection with a small clinical sample size (<100) and using the C-prM gene [38] or serotype-specific regions of the 3′ untranslated region (UTR) [36,37] as the amplification targets. In the present study, we described an improved method for the development and application of the RT-LAMP assay for the detection of DENV infection in freshly obtained dengue-suspected patient samples in actual clinical laboratory setting of a hospital in dengue endemic environment.

## Methods

### Dengue viruses

A total of 11 reference DENV strains (four strains of DENV-1, genotype I, II, III and sylvatic; two strains of DENV-2, Asian I and cosmopolitan; three strains of DENV-3, genotype I, II and III; two strains of DENV-4, subgenotype IIa and IIb) were used in this study [30,39-41]. All the viruses were archived in the University Malaya Medical Centre (UMMC) Diagnostic Virology Laboratory repository. The isolates at passage 1 were used to inoculate C6/36 (*Aedes albopictus*) mosquito cells for one week and virus RNA was extracted from the infected cell culture supernatant.

### Clinical samples

The study obtained ethics approval from the UMMC Medical Ethics Committee (Ethics Committee/IRB Reference Number: 908.11). The study was conducted during the period from October to December 2012. A total of 305 serum samples from patients clinically suspected with DENV infection were obtained. All clinical samples were taken as part of standard patient care. The samples were divided in the laboratory to be used for routine diagnosis by serological assays and evaluation of the RT-LAMP assay developed in this study.

### RNA extraction

Total RNA was extracted from 140 μl of infected culture supernatant or patient serum samples using QIAamp Viral RNA Mini Kit (Qiagen, Germany), following the manufacturer’s protocol. All RNA extraction was done manually. The RNA was eluted in 60 μl of nuclease-free water and stored at −80°C until needed.

### Design of DENV-specific RT-LAMP assay primers

The DENV-specific primers used for RT-LAMP assay were designed from the 3′UTR of the DENV genome. The 3′UTR nucleotide sequences of each DENV serotypes were retrieved from GenBank. Multiple sequence alignment of the 3′UTR sequences was performed using Clustal X 2.0 [42]. A set of nine primers comprising four outer, four inner, and one loop primers that recognize seven distinct regions on the target sequence was designed according to the criteria described by Notomi *et al.*[33] (Table 1 and Additional file 1). The coverage of the RT-LAMP primers was validated by evaluating the assay using viral RNA extracted from the different DENV strains, as described above.

**Table 1:** RT-LAMP primers used for the rapid detection of DENV

**Primer**^**a,b**^	**Sequence (5′ → 3′)**
F3/134	CAAACCGTGCTGCCTGT
F3/2	TGAGTAAACTATGCAGCCTGT
B3/123	ACCTGTTGATTCAACAGCACC
B3/4	ACCTGTTGGATCAACAACACC
FIP/123	AGGGGTCTCCTCTAACCRCTAGTCTTTCAAACCRTGGAAGCTGTACGC
FIP/4	AGGGGTCTCCTCTAACCRCTAGTCTTTTTTGCCACGGAAGCTGTACGC
BIP/123	ACAGCATATTGACGCTGGGARAGACGTTCTGTGCCTGGAATGATGCTG
BIP/4	ACAGCATATTGACGCTGGGARAGACGCTCTGTGCCTGGATTGATGTTG
BLP/1234	CAGAGATCCTGCTGTCTC

^a^*F3* forward outer primer, *B3* backward outer primer, *FIP* forward inner primer, *BIP* backward inner primer, *BLP* backward loop primer.

^b^Number (1–4) after the slash (/) represents DENV serotype (e.g. F3/134, forward outer primer for DENV-1, -3 and −4).

### RT-LAMP assay

The RT-LAMP was performed in a final reaction volume of 25 μl using a Loopamp RNA Amplification Kit (Eiken Chemical Co. Ltd., Japan) added with 20 pmol each of inner primers FIP/123, FIP/4, BIP/123, and BIP/4; 2.5 pmol each of outer primers F3/134, F3/2, B3/123, and B3/4; 20 pmol of loop primer BLP/1234; 1 μl of Fluorescent Detection Reagent (Eiken Chemical Co. Ltd., Japan); and 5 μl of the extracted RNA template. A positive control using 1000 copies (determined by qRT-PCR) of DENV RNA extracted from culture supernatant and a negative control (nuclease-free water) were included in each run. The RT-LAMP reactions were incubated at 63°C for 80 min and inactivated at 80°C for 5 min in LA-500 Loopamp real-time turbidimeter (Eiken Chemical Co. Ltd., Japan). The turbidity of RT-LAMP reaction was spectrophotometrically recorded at 650 nm every 6 s. The threshold time (Tt) value for positivity by RT-LAMP was determined when the turbidity increased above the threshold value, which was fixed at 0.07 absorbance units. The RT-LAMP amplification was also visually monitored for color change. Positive reaction turned the reaction mix green and fluoresces under the white light and UV irradiation, respectively. The reaction mix remained orange and non-fluorescent in the absence of amplification.

### Specificity of RT-LAMP assay

The specificity of the RT-LAMP amplification was assessed by single site restriction enzyme digestion of the amplified DNA fragments using BanII [36]. Following overnight digestion at 37°C, the undigested and digested RT-LAMP-amplified DNA fragments were electrophoresed on a 2% agarose gel in Tris-acetate-EDTA buffer. The gel was stained with GelRed (Biotium Inc., US) and visualized using a Gel Doc 2000 (Bio-Rad Laboratories, Inc., US). The digested DNA fragments were sequenced using the loop primer BLP/1234. The specificity of the DENV RT-LAMP primers was evaluated against another three closely related arboviruses common in the region; Japanese encephalitis virus (JEV), Chikungunya virus (CHIKV), and Sindbis virus (SINV).

### Sensitivity of RT-LAMP assay

The sensitivity of RT-LAMP assay was assessed using a panel of serially diluted viral RNA (1000, 100, 60, and 10 copy numbers) extracted from culture supernatant. The sensitivity test of RT-LAMP was repeated twelve times. The viral RNA used was quantitated using the *genesig* Real-Time qRT-PCR DENV Detection Kit (PrimerDesign Ltd., UK). The qRT-PCR assay standard plot, ranged from 10 to 10^6^ RNA copies, was made by preparing a 10-fold serial dilution of the *genesig* DENV RNA standard. The *genesig* DENV RNA standard was a synthetic RNA template with known copy number. The qRT-PCR was performed in a final volume of 20 μl containing 10 μl of real time master mix, 1 μl of probe/primer mix, 4 μl of nuclease-free water, and 5 μl of diluted RNA. Quantitative PCR measurement was performed using StepOnePlus real time PCR system (Applied Biosystems, USA) according to the following condition: 10 min at 55°C, 8 min at 95°C followed by 50 cycles of amplification (10 s at 95°C, 60 s at 60°C). Raw data was analyzed with StepOne Software v2.2.1 to determine copy number based on the threshold cycles (Ct). The efficiency of the qRT-PCR was measured from the slope of standard curve.

### Evaluation of RT-LAMP assay

The clinical applicability of a single-tube RT-LAMP assay for detection of DENV RNA was assessed in a total of 305 serum samples freshly obtained from dengue-suspected patients. The qRT-PCR, as described above, was used as a reference assay for the detection of DENV RNA in the samples. In addition, the samples were simultaneously screened for the presence of anti-dengue IgM using the SD Dengue IgM Capture ELISA kit (Standard Diagnosis Inc., Korea). The IgM-negative samples were further screened for the presence of anti-dengue IgG using the SD Dengue IgG Capture ELISA kit (Standard Diagnosis Inc., Korea). The test results of RT-LAMP, qRT-PCR, and ELISA were compared. Acute DENV infection was confirmed by the positive detection of anti-DENV IgM by ELISA and/or the presence of DENV RNA detected by qRT-PCR assay. Serum sample tested positive only for anti-DENV IgG was identified as indicative of past DENV infection.

In this study, the results obtained by molecular diagnosis of dengue were not used for the standard patient care as the molecular assay was still being developed. The qRT-PCR was used only as research tool in the reference laboratory. Currently, only the serological methods were approved for routine clinical diagnosis of dengue at the UMMC.

### Statistical analysis

All statistical analysis was performed using IBM SPSS Statistics, version 21 (IBM Corporation, New York, United States). The degree of agreement between RT-LAMP and qRT-PCR test results was measured by kappa value (κ). Chi-square test (McNemar’s exact test, two-tailed) was performed to evaluate and compare the sensitivity of all molecular and serological methods used. In the present study, the p-value <0.001 was used to suggest significant results. The diagnostic performance of RT-LAMP assay as compared to qRT-PCR was calculated using web-based CEBM Statistics Calculator (http://ktclearinghouse.ca/cebm/toolbox/statscalc).

## Results

### Design of DENV-specific RT-LAMP assay primers

**Figure 1:**
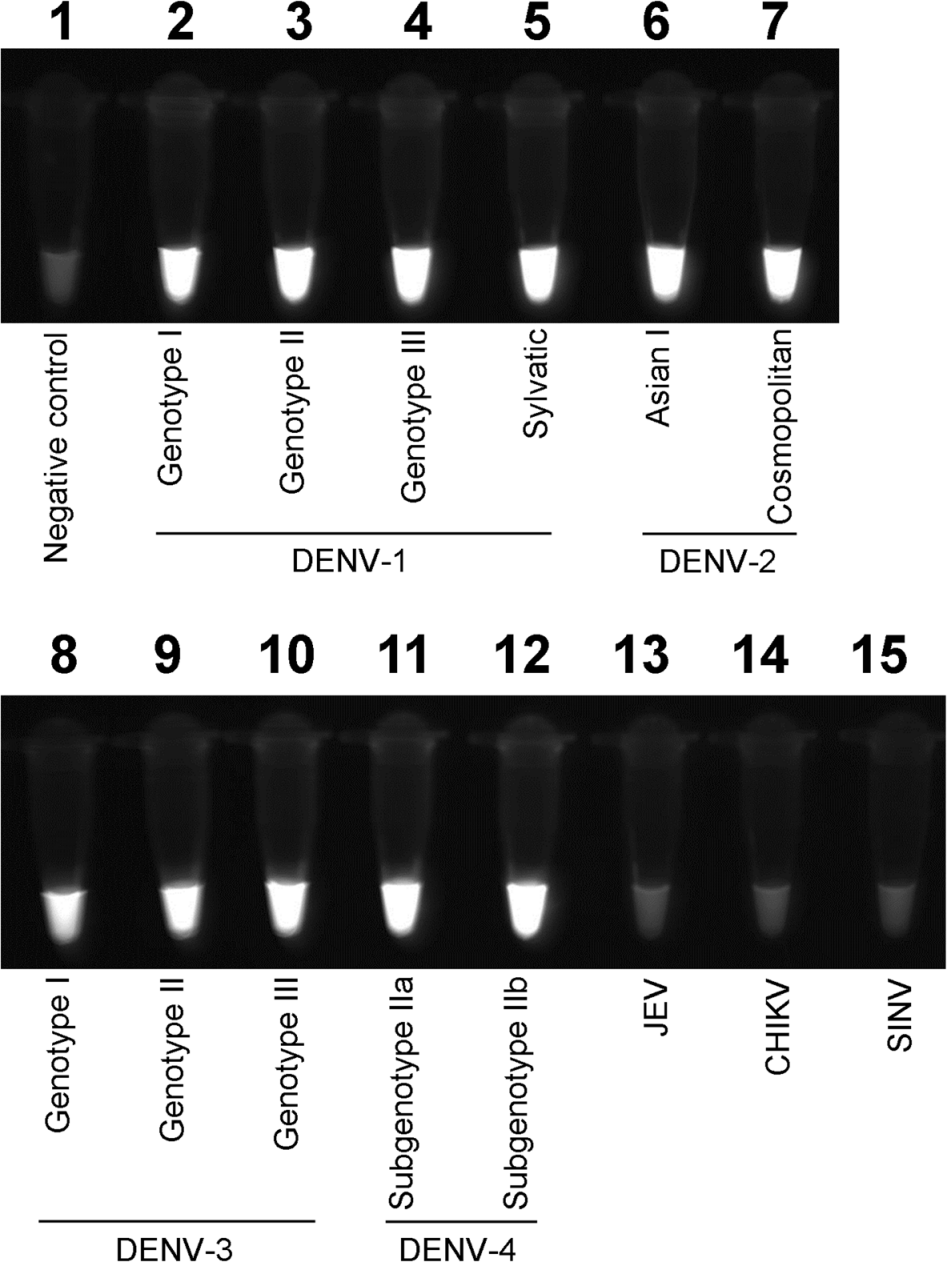
**Visual observation of fluorescence in RT-LAMP reactions under UV light.** Tube 1, negative control; tube 2–5, DENV-1 genotype I, II, III and sylvatic, respectively; tube 6–7, DENV-2 Asian I and cosmopolitan, respectively; tube 8–10, DENV-3 genotype I, II and III, respectively; tube 11–12, DENV-4 subgenotype IIa and IIb, respectively; tube 13–15, JEV, CHIKV and SINV, respectively.

In order to develop the single-tube RT-LAMP assay for detection of all four DENV serotypes, a set of nine primers comprising four outer (F3/134, F3/2, B3/123, and B3/4), four inner (FIP/123, FIP/4, BIP/123, and BIP/4), and one loop primers (BLP/1234) were designed. This was done using the alignment of the conserved 3′UTR regions of DENV genome (Table 1 and Additional file 1). Results shown in Figure 1 suggested that the RT-LAMP primers detected the entire panel of 11 available reference DENV strains.

### Specificity of RT-LAMP assay

**Figure 2:**
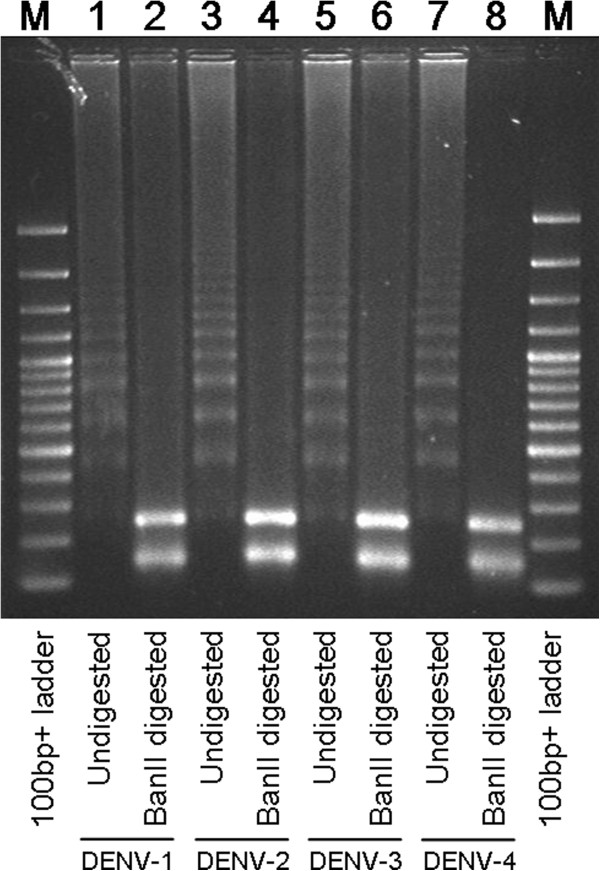
**Confirmation of RT-LAMP amplification of DENV.** Restriction enzyme digestion of DENV-specific RT-LAMP-amplified DNA fragments were performed using BanII. The DNA fragments were separated on a 2% agarose gel. Lane M, 100-bp plus DNA ladder; lane 1, DENV-1 RT-LAMP assay amplification; lane 2, BanII digestion of DENV-1 RT-LAMP-amplified DNA fragments, 129 bp and 233 bp; lane 3, DENV-2 RT-LAMP assay amplification; lane 4, BanII digestion of DENV-2 RT-LAMP-amplified DNA fragments, 135 bp and 231 bp; lane 5, DENV-3 RT-LAMP assay amplification; lane 6, BanII digestion of DENV-3 RT-LAMP-amplified DNA fragments, 129 bp and 231 bp; lane 7, DENV-4 RT-LAMP assay amplification; lane 8, BanII digestion of DENV-4 RT-LAMP-amplified DNA fragments, 141 bp and 231 bp.

No cross-reactivity of the RT-Lamp assay was observed with all other three closely related arboviruses common in the region, including JEV, CHIKV and SINV (Figure 1). The specificity of the assay was also verified by restriction enzyme BanII digestion on the amplified DNA fragments of all four DENV serotypes. As shown in Figure 2, the undigested and digested DNA fragments were observed as ladder-like and two-band patterns, respectively [36,37]. The sizes of the digested DNA fragments were in agreement with the expected size for each serotype; 129 bp and 233 bp for DENV-1, 135 bp and 231 bp for DENV-2, 129 bp and 231 bp for DENV-3, as well as 141 bp and 231 bp for DENV-4. Nucleotide sequencing of the 231 bp and 233 bp digested DNA fragments confirmed that the RT-LAMP amplified nucleotide sequences were specific to DENV (data not shown).

### Sensitivity of RT-LAMP assay

**Figure 3:**
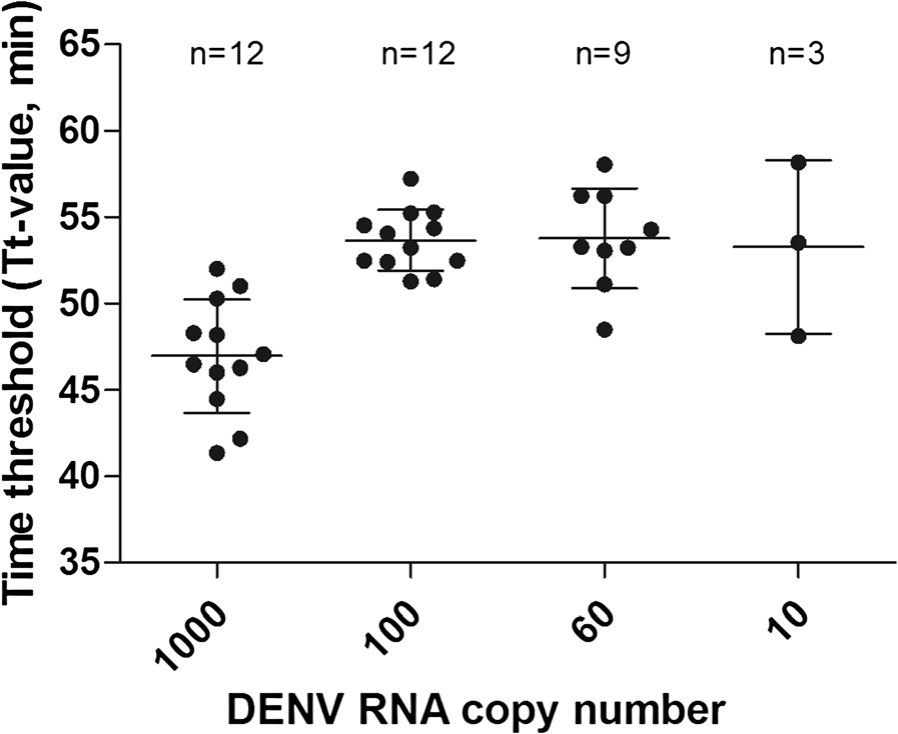
**Time threshold of positivity for RT-LAMP assays of serially diluted DENV RNA.** The mean of Tt-values was calculated with available positive results out of twelve replicates. Error bars indicate the standard deviations of Tt-values from the mean.

The sensitivity of RT-LAMP assay was determined by repeated independent testing on a panel of serially diluted viral RNA extracted from culture supernatant with known copy number (Figure 3). The number of positive detection by RT-LAMP assays (n = 12) for the DENV RNA with copy numbers of 1000, 100, 60, and 10 were 100% (12 of 12), 100% (12 of 12), 75% (9 of 12), and 25% (3 of 12), respectively, with the mean time threshold (Tt) of 46.97 ± 2.28 min, 53.67 ± 1.77 min, 53.78 ± 2.89 min, 53.28 ± 5.04 min, respectively. The RT-LAMP assay detected up to 10 copies of RNA but 100% (12 of 12) reproducibility was achieved with at least 100 copies of RNA.

### Evaluation of RT-LAMP assay

The RT-LAMP assay for detection of DENV RNA was evaluated by testing on a total of 305 fresh serum samples of clinically dengue-suspected patients. The findings of RT-LAMP were compared to those obtained by qRT-PCR. Of the 305 samples, 282 were first serum samples while 23 were second samples collected at 1 to 5 days from the first sample. Out of 305 samples, acute dengue infection was confirmed in 171 (56.1%) by either qRT-PCR or dengue IgM ELISA, or both (Table 2). Five out of the 171 samples (2.9%) were identified as secondary DENV infection as virus RNA and dengue-specific IgG were detected in the absence of dengue IgM. Seven samples were identified as past DENV infection as only dengue IgG was tested positive.

**Table 2:** Summary of dengue detection in serum samples from clinically dengue-suspected patients in UMMC using RT-LAMP, qRT-PCR, IgM and IgG ELISA

		**IgM ELISA**		**IgG ELISA**^**b**^		
		**Positive**	**Negative**	**Positive**	**Negative**	**Not-tested**
**Assays**	**Results**^**c**^	**n (%)**	**n (%)**	**n (%)**	**n (%)**	**n (%)**
RT-LAMP	Positive	23 (30.7)	52 (69.3)	5 (6.7)	40 (53.3)	30 (40.0)
	(n = 75^d^)					
	Negative	93 (40.4)	137 (59.6)	7^f^ (3.1)	130 (56.5)	93 (40.4)
	(n = 230)					
qRT-PCR	Positive	25^a^ (31.3)	55^a^ (68.7)	5 (6.3)	43 (53.7)	32 (40.0)
	(n = 80^e^)					
	Negative	91^a^ (40.4)	134 (59.6)	7^f^ (3.1)	127 (56.5)	91 (40.4)
	(n = 225)					

^a^Acute DENV infection was confirmed in 171 samples.

^b^Dengue IgG ELISA was only performed on 182 (out of 189) dengue IgM negative samples.

^c^Agreement of results between RT-LAMP and qRT-PCR, κ = 0.939.

^d^One of RT-LAMP positive samples were tested negative by qRT-PCR

^e^Six of qRT-PCR positive samples were tested negative by RT-LAMP.

^f^DENV RNA was tested negative by both RT-LAMP and qRT-PCR in 7 samples.

**Figure 4:**
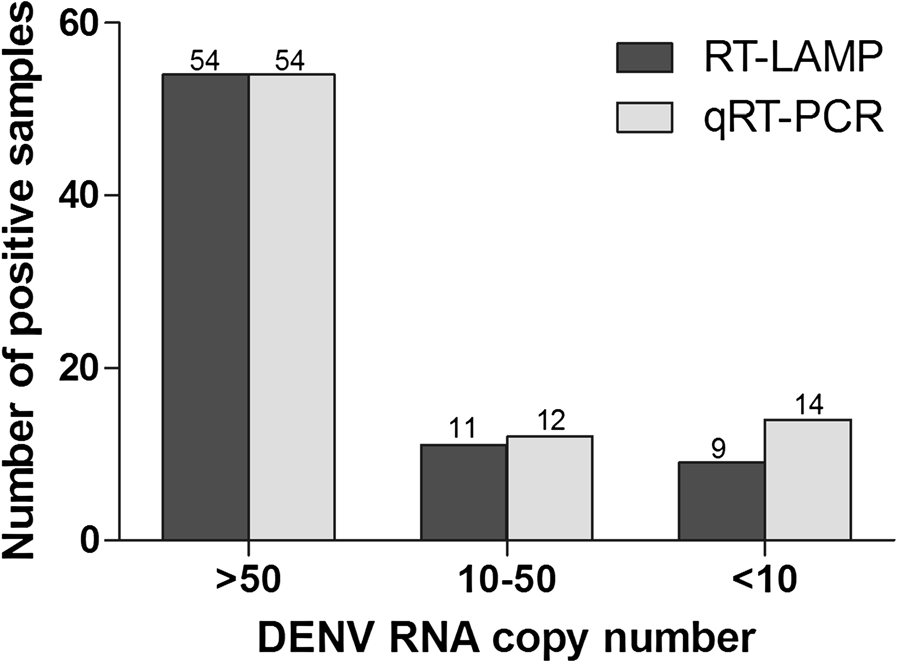
**Number of clinical samples positive by RT-LAMP and qRT-PCR.** Six samples were tested positive for DENV RNA by qRT-PCR but negative by RT-LAMP. One sample positive by RT-LAMP but negative by qRT-PCR was excluded.

**Figure 5:**
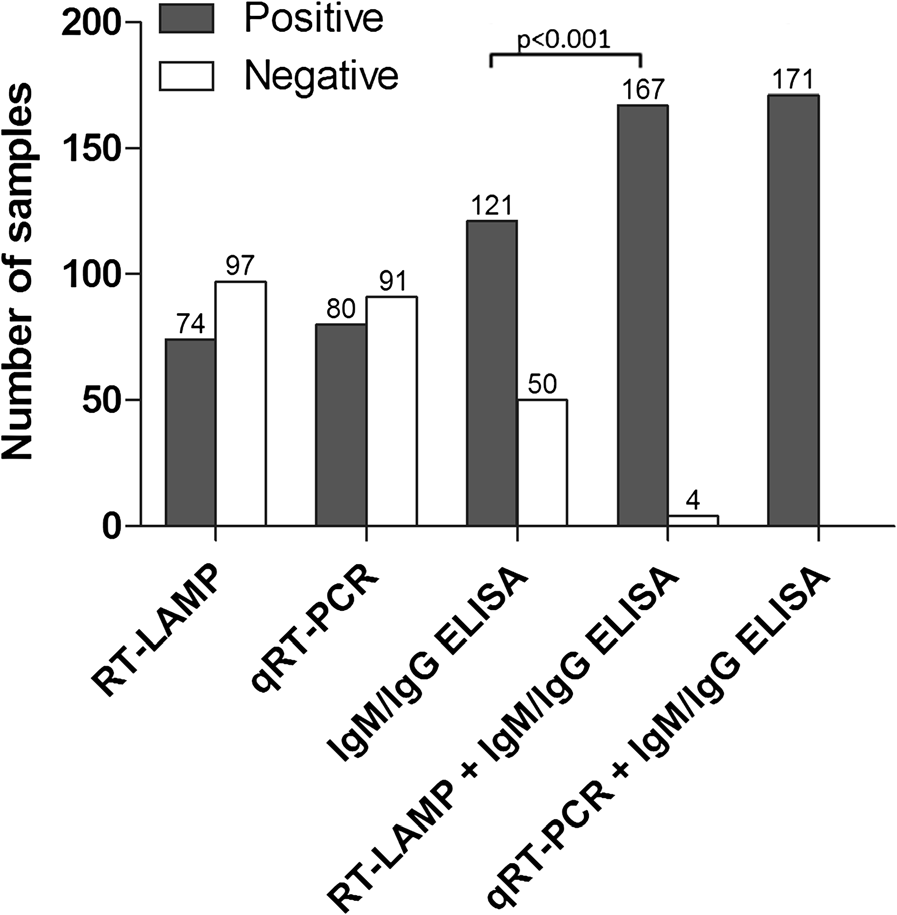
**Sensitivity of dengue diagnostic methods against laboratory-confirmed dengue samples (n = 171).** Sensitivity between groups was compared using McNemar’s exact test (two-tailed).

The diagnostic performance of the RT-LAMP in comparison with qRT-PCR assay is summarized in Table 3. The RT-LAMP assay detected DENV genome in 74 of 171 (43.3%) of the acute dengue samples compared to 80 of 171 (46.8%) by qRT-PCR assay (Table 2). These two methods, however, showed high concordance with kappa value of 0.939 (p < 0.001). DENV RNA was tested negative in 6 samples by RT-LAMP but positive by qRT-PCR. All of these samples contained <50 copies of RNA per reaction (Figure 4). In this study, one sample that was tested positive by RT-LAMP but negative by qRT-PCR or serological methods was considered as false positive. The combination of RT-LAMP with the dengue IgM and IgG ELISA resulted in a significant increase (p < 0.001) in sensitivity to 97.7% (167 of 171) in comparison to using dengue IgM and IgG ELISA alone which had sensitivity of 70.8% (121 of 171) (Figure 5).

**Table 3:** Diagnostic performance of RT-LAMP assay against qRT-PCR assay in serum samples from clinically dengue-suspected patients in UMMC

		**qRT-PCR**		**Sensitivity% (95%CI)**	**Specificity% (95% CI)**	**PPV% (95% CI)**	**NPV% (95% CI)**
**Assay**	**Results**	**Pos**	**Neg**				
RT-LAMP	Pos	74	1	92.5 (84.6-96.5)	99.6 (97.5-99.9)	98.7 (92.8-99.8)	97.4 (94.4-98.8)
	Neg	6	224				

*PPV* positive predicted value, *NPV* negative predicted value, *CI* confidence interval, *Pos* positive, *Neg* negative.

## Discussion

In the present study, a single-tube RT-LAMP assay was developed with a set of nine primers targeted at the highly conserved regions of DENV 3′UTR of the reported global DENV strains. We demonstrated that the RT-LAMP assay detected a panel of DENV strains from different DENV genotypes circulating in Malaysia and many other dengue endemic countries in Southeast Asia [30,39-41]. There was no cross reactivity of RT-LAMP with other closely related arboviruses. The RT-LAMP assay was as sensitive as the qRT-PCR for DENV detection in the viremic serum samples. In this study, the RT-LAMP assay is positioned as a complementary tool for the routine serology test in a reference diagnostic laboratory.

The RT-LAMP assay developed here differs from those reported earlier in at least two major areas [36-38]. Parida *et al.* (2005) developed a four-tube RT-LAMP assay which employed serotype-specific primers targeting the proximal half of the 3′UTR of DENV genome [36]. The four-tube reaction system required four separate reactions to be performed for each sample. Therefore, it is not cost-effective for routine diagnosis particularly in the hospital setting where the interest is simply in knowing if the patient has dengue. Recently, Lu *et al.* developed a single-tube reaction system for the detection of DENV infection using RT-LAMP primers derived from the C-prM gene [38]. The C-prM gene, however, was relatively less conserved among all four DENV serotypes (inter-serotype) in comparison to the 3′UTR (unpublished data). Thus, the primers designed based on the C-prM gene may not give a good coverage for all four DENV serotypes and their genotypes. In many dengue hyperendemic countries, multiple DENV genotypes of all four serotypes co-circulate [30,39-41]. In our study, we used the combination of primers targeting the highly conserved region of DENV 3′UTR. The nine primers were generated to be useful to accommodate the viral diversity present in the region. We showed here that all the 11 different reference DENV strains from 10 DENV genotypes were detected with the RT-LAMP primers designed in the study.

The RT-LAMP assay developed in this study has a detection limit of at least 100 copies of viral RNA (Figure 3). We defined the detection limit in this study as the lowest amount of template detectable with 100% reproducibility throughout the repeated testing. Other similar studies have reported detection limit of lower than 100 copies but with lack of information on the confidence level [37,38]. Our RT-LAMP assay was able to detect up to as few as 10 copies of viral RNA, but with reduced reproducibility. The viral load was inversely proportional to the antibody titer according to the day of onset of fever. Low viral load (<100 viruses) is usually accompanied with the rise of antibody titer [43,44]. The detection of DENV using RT-LAMP regardless of the viral load could be completed within 1 hour making the assay a rapid tool for early dengue diagnosis.

Here, the RT-LAMP assay for DENV detection was prospectively evaluated for the first time with a large number of clinical serum samples (n = 305) from dengue-suspected patients. The performance of the RT-LAMP assay was validated by simultaneous testing of the samples using real time qRT-PCR, which is known as the most sensitive and specific method for the detection of viral RNA [45]. In our study, both the RT-LAMP and qRT-PCR assays showed comparable sensitivity for the detection of DENV in patients’ sera (κ = 0.939), even though the sensitivity of RT-LAMP was slightly lower than that of qRT-PCR. Similar findings have been reported in several other studies for the evaluation of LAMP assay when compared against the qPCR [46,47]. The RT-LAMP assay in this study gave possibly six false negative and one false positive results. The false negative detection could be caused by low viral load, below the detection limit of RT-LAMP. However, this could also reflect false positive detection of the qRT-PCR. This could be verified by culturing the samples for virus isolation. Similarly, the false positive RT-LAMP result could be a true positive but need to be verified by virus isolation.

Currently, in most dengue endemic region where resources are limited, early diagnosis of dengue relies very much on the clinical presentations. A number of other infections, however, can present with almost similar features especially during the early febrile phase. Rapid confirmation of dengue is important to ensure proper management of patients and appropriate outbreak prevention measures. Currently, serological assay is the most common method used to confirm DENV infection. In our study, using actual clinical samples when febrile dengue patients first visit to the hospital, 46.8% (80 of 171) of the specimen received indicated that the patients were viremic with the absence of IgM or IgG (Figure 5). The RT-LAMP or qRT-PCR, when used in combination with ELISA, increased the diagnostic coverage of febrile dengue patient to more than 97%. The early detection of viremic individuals allows for immediate intervention to prevent further spread of dengue by encouraging the patient to take precaution to prevent from mosquito bites. In addition, it allows the patient to have sufficient warning to immediately come back to hospital if warning signs of impending severe dengue develops.

## Conclusions

The RT-LAMP assay developed in our study showed high sensitivity and specificity comparable to qRT-PCR for detection of DENV in clinical samples. The single-tube RT-LAMP assay utilized a primer set with coverage for all the reported DENV strains particularly those common in the region. Implementation of the RT-LAMP assay into routine dengue diagnosis to complement the antibody detection would greatly enhance the diagnostic coverage of suspected dengue cases without the need for costly equipment and reagents. The RT-LAMP assay developed in this study is sensitive, specific and simple to perform, and in combination with the ELISA is a promising detection tool for early dengue diagnosis in rural clinics and field situation where resources are limited.

## Competing interests

All authors have no competing interests.

## Authors’ contributions

BTT, SSS, KKT, JJ, MBD, and SAB conceived the study and participated in its design and coordination. BTT designed the RT-LAMP primers. BTT and SSS performed sample preparation and RNA extraction. BTT, SSS, KKT, PSH, and RME performed the RT-LAMP, qRT-PCR and ELISA assays. BTT, SSS, KKT, and PSH analyzed the results. SSS carried out the statistical analysis. BTT, SSS and SAB drafted the manuscript. SAB is the principle investigator of this study. All authors read and approved the final manuscript.

## Pre-publication history

The pre-publication history for this paper can be accessed here:

http://www.biomedcentral.com/1471-2334/13/387/prepub

## Supplementary Material

Additional file 1: Figure S1 Map of RT-LAMP primers in alignment with the DENV 3’UTR consensus sequences. The arrows indicate the orientation of primers in 5′ to 3′ direction. Click here for file
