# Supplementary figures and images for: Detection of dengue viruses using reverse transcription-loop-mediated isothermal amplification
Source: BMC Infect Dis. 2013 Aug 21;13:387. doi: 10.1186/1471-2334-13-387 (PMC3846474; doi:10.1186/1471-2334-13-387)

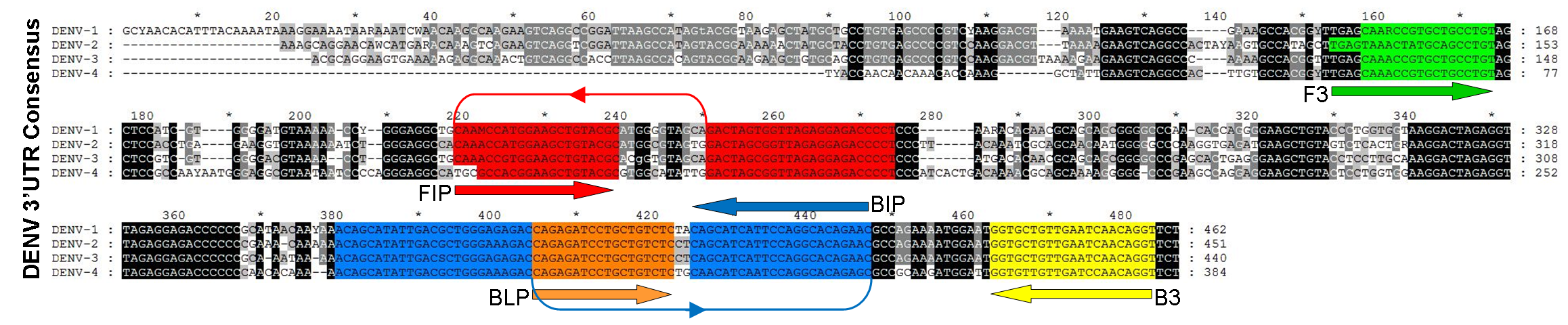

Supplement: Additional file 1: Figure S1 — Map of RT-LAMP primers in alignment with the DENV 3’UTR consensus sequences. The arrows indicate the orientation of primers in 5′ to 3′ direction. [file 1471-2334-13-387-S1.tiff]
